# Is Obesity Associated With Dental Caries in Primary Dentition? Findings From a Birth Cohort in Southern Brazil

**DOI:** 10.1111/cdoe.70056

**Published:** 2026-02-20

**Authors:** Yorrana Martins Corrêa, Cinthia Fonseca Araujo, Mariana Silveira Echeverria, Andréa Dâmaso Bertoldi, Geert J. M. G. van der Heijden, Flávio Fernando Demarco, Helena Silveira Schuch

**Affiliations:** ^1^ Graduate Program in Dentistry Federal University of Pelotas Pelotas Rio Grande do Sul Brazil; ^2^ Graduate Program in Epidemiology Federal University of Pelotas Pelotas Rio Grande do Sul Brazil; ^3^ Oral Public Health Department ACTA, Academic Center for Dentistry, University of Amsterdam Amsterdam the Netherlands; ^4^ Population Oral Health, School of Dentistry The University of Queensland Herston Queensland Australia

**Keywords:** child, cohort studies, deciduous, dental caries, obesity, tooth

## Abstract

**Objectives:**

Nutritional disorders and dental caries share common risk factors, including diet and socioeconomic position, but the association between these two conditions remains unclear. The aim of this study was to investigate the potential association between overweight/obesity and the occurrence of dental caries in primary dentition.

**Methods:**

This longitudinal study, conducted using data from the 2015 Pelotas Birth Cohort, utilised information collected at birth, 24 months and 4 years of age. The exposure variable was the obesity collected by BMI (body mass index), at 24 months, using anthropometric measures (weight and height) and classified according to WHO guidelines. Covariates included sex, child's and maternal age, socioeconomic factors (family income and maternal education) and sugar consumption data. The outcome was dental caries at the age of four, assessed using several variables: early childhood caries (ECC), encompassing all activity including white spots and restorations; severe early childhood caries (S‐ECC), which refers exclusively to cavitated lesions; and the dmft (Decayed, Missing and Filled Teeth) index. Descriptive analysis was carried out and regression models were tested.

**Results:**

From the 4275 eligible participants, 3374 children (50.5% boys and 49.5% girls) composed the analytical sample. At 24 months, 2474 (73.3%) children were healthy weight (95% CI 71.8; 74.8) and 900 (26.7%) presented overweight/obesity (18.8% overweight and 7.9% obese). Concerning dental caries at the age of four, 37.6% had ECC (including white spots and restorations) and 21.4% had S‐ECC. Among children classified as healthy weight at 24 months, 38.6% presented ECC at 4 years of age. Among obese children, the prevalence of ECC was 34.8%. A similar pattern was observed for S‐ECC: the prevalence was 22.0% among healthy weight children, 19.9% among overweight and 19.1% among obese children. The Poisson regression analysis adjusting for confounding factors showed no difference between groups.

**Conclusions:**

In conclusion, this cohort study in Brazilian children at the age of four did not observe meaningful associations between overweight/obesity and dental caries in primary dentition. The findings suggest that obesity in childhood should not be considered a risk factor for caries development in children.

## 1 Introduction

Dental caries remains the most common chronic disease in childhood, affecting an estimated 514 million children globally [1]. It places a significant economic burden on families and health systems and negatively impacts children's quality of life [2]. While the global prevalence of oral diseases has slightly declined in recent decades, rates continue to rise in many low‐ and middle‐income countries, driven by increasing urbanisation and changes in living conditions [3]. In Brazil, the most recent national oral health survey reported that 41.18% of 5‐year‐old children had experienced dental caries [4].

Sugar consumption is the causal condition of caries development, and both the amount and frequency of ingestion are closely related to disease burden [5]. In recent years, global sucrose production and consumption have increased, contributing to a higher prevalence of sucrose‐related conditions such as dental caries, obesity and diabetes [2, 6].

In Brazil, overweight and obesity are nutritional disorders more prevalent than undernutrition [7]. Obesity is a chronic condition associated with increased morbidity and mortality, and it is a known risk factor for systemic diseases, including Type 2 Diabetes Mellitus, hypertension and cancer [8]. Among Brazilian children under 5 years of age, approximately 10% are affected, with regional variations observed among those aged 5–10 years [9, 10]. Globally, the rise in obesity since 1975 has outpaced changes in the prevalence of moderate and severe underweight [11].

Dental caries and obesity share several common risk factors, such as unhealthy diet and lower socioeconomic position [12]. However, evidence regarding the association between the two conditions remains inconsistent. Some studies report a higher prevalence of dental caries among obese individuals, reflecting shared etiological pathways involving sugar consumption [13, 14]. Conversely, other studies have found greated caries prevalence among individuals with normal weight [15, 16, 17, 18]. Additionally, after adjusting their estimates for covariates, such as race and ethnicity, familial structure and access to dental services, some studies found no significant association between obesity and dental caries [19, 20, 21]. Systematic reviews have also failed to establish a consistent relationship between these conditions [22], including among Brazilian children and adolescents [23]. These findings highlight the need for well‐designed longitudinal studies to clarify whether early‐life nutritional status influences the development of dental caries [22], as this is the most suitable design in observational research to investigate if different exposures during the life cycle could impact the occurrence of a given outcome [24].

Considering the high prevalence of dental caries and nutritional disorders and their potential impact on children's well‐being, the aim of this study was to estimate the extent to which overweight/obesity at 24 months of age is associated with the presence of dental caries at age four in children from the 2015 Pelotas Birth Cohort.

## 2 Methods

The present research is structured according to the STROBE (STrengthening the Report of OBservational studies in Epidemiology) guidelines [25].

### 2.1 Study Design

This study used data from the 2015 Pelotas Birth Cohort Study. Pelotas is a medium‐sized city, with around 330 000 people, located in southern Brazil [26].

During the entire year of 2015, a research team conducted daily visits to all hospitals in Pelotas, to identify live‐born children whose mothers lived in the city's urban area. Based on the eligibility criteria, 4387 births were recorded, of which 54 were stillborn, 51 were refusals and seven were not captured by the study teams. Then, the 2015 Birth Cohort was composed of 4275 children. Besides the baseline, seven follow‐ups have been performed. The present study uses data from the perinatal, 24‐month follow‐up (*n* = 4018; 95.4%) and 4‐year follow‐up (*n* = 4010; 95.4%). The variables used were collected using standardised questionnaires, anthropometric measurements and clinical oral examinations. More detailed information about the 2015 Birth Cohort methodology can be obtained elsewhere [27, 28].

At each follow‐up, interviewers received both theoretical and practical training. A pilot study and quality control measures were conducted for each follow‐up to ensure accuracy and consistency. The clinical oral examination at the age of four was performed in 3645 children (91.1% of the children evaluated at that follow‐up) at the Epidemiological Research Center by a team of 12 trained and calibrated dentists. Parents signed an informed consent form. The inter‐examiner agreement on dental caries measured by the weighted Kappa statistic was 0.91.

### 2.2 Variables

#### 2.2.1 Exposure

Obesity was used as the exposure variable, measured by the weight and height of the child at 24 months. The anthropometric measures were collected using standard measurements. The weight was collected in kilograms (kg) using a scale SECA model 803, with 100 g precision and a capacity of 1050 kg and the height was assessed using a portable anthropometer (SANNY model ES2000), with an amplitude of 20 to 105 cm and precision of 0.5 cm. The overweight/obesity case definition was calculated using the formula [BMI = weight/(height × height)], using the criteria established by the Multicentric Reference Study from the World Health Organization (WHO). They present the reference percentiles of BMI for children, according to age and sex. Following such criteria, children below percentile were considered to have low BMI, those between percentiles three and < 85 as healthy weight, children with BMI values between ≥ 85 and < 97 were classified as overweight, and those with BMI percentiles ≥ 97 were identified as obese [29]. Undernutrition was not considered as a separate category due to the low prevalence in the sample (51 participants at 24 months, 1.4%), and these children were grouped with healthy weight children.

#### 2.2.2 Outcome

The outcome of this study was dental caries in all primary teeth, which were clinically evaluated only at the age of four. The examination was carried out by trained and calibrated dentists, following the biosafety guidelines from WHO. Personal protective equipment and a headlamp were used to perform the examination. For caries evaluation, the Simplified International Caries Detection and Assessment System (ICDAS) was used [30]. This simplified score comprises a smaller number of codes, based on the severity of the lesion: sound tooth, initial stage, moderate stage and advanced stage. Code zero was used for healthy teeth. Code one was used for initial carious lesions, presented as white spots, without signals of enamel cavitation. Code two was used for caries in the moderate stage, comprising white spots with enamel cavitation and lesions with dentine shadows. Code three was used for caries lesions in an advanced stage, with cavitation in dentine.

For analysis purposes, in this study, the dental caries outcome is evaluated through three variables: (a) early childhood caries (ECC), which encompasses the entire experience of dental caries, including evidence of initial lesions or the presence of dental filling, such as white spots and restorations; (b) severe early childhood caries (S‐ECC), which refers to the severity of caries, specifically involving cavitated lesions; and (c) the dmft index, which provides information on the Decayed, Missing and Filled Teeth, assessing the extent of dental caries in the individual [31].

### 2.3 Covariates

Maternal level of education and family income, both collected at the perinatal period, were used as covariates. Maternal education was collected as the number of years of formal education completed, with options “zero to four years”, “five to eight”, “nine to eleven” or “12 years or more”. Family income was collected as the sum of earnings of all family members in the last month, in Brazilian currency (Real). For analytical purposes, maternal education was dichotomized as up to 11 years versus 12 or more years of formal education, which represents, in Brazil, incomplete high school versus completed high school or more. Family income was dichotomized as less than two monthly minimum wages (MW) versus two or more MW.

Additionally, other variables used as covariates included the child's sex (male or female), maternal age at birth (categorised as < 20, 20–34 and ≥ 35), the child's age at follow‐up, and sugar consumption at 24 months. Sugar consumption was assessed through the intake of sugary foods and beverages, such as bottled, boxed, or powdered juice, bottled or boxed coconut water, soft drinks, sweet cookies, candies, lollipops, chewing gums, chocolates, jelly and any sugar in the bottled drinks. The list of sugary products included 6 groups of products. For analytical purposes, sugar consumption was dichotomized into low versus high consumption, based on the median number of sugary products consumed by the participants. Children who consumed up to three items from the list were coded as “low sugar consumption” and those children that consumed four or more items were coded as “high sugar consumption”.

### 2.4 Statistical Analysis

Data analysis was performed using the Stata Statistical Software, version 17.0. For analytical purposes, dental caries outcomes were assessed ranging from early childhood caries, which refers to the experience of dental caries, to severe early childhood caries, which considers cavitated lesions. The statistical analysis encompassed descriptive analyses of relative and absolute frequencies of variables, and the distribution of outcomes according to BMI, overall and stratified by socioeconomic indicators, using the chi‐square test. A Poisson regression was performed to evaluate the association between exposure and outcome adjusted for the following confounding factors: child's sex, maternal age, maternal education and family income, all collected at the perinatal interview, as well as the child's age at follow‐up and sugar consumption at 24 months. The selection of confounders was informed by prior literature on social determinants, obesity and dental caries, as well as theoretical relevance to the exposure–outcome relationship. In the regression models, overweight and obese children were grouped into a single category, given the small number of obese children in the sample (267 children, 7.9%).

In addition, inequity were assessed using the Equiplot Creator Tool, developed by the ICEH, International Center for Equity in Health [32]. These graphics not only provide insights into the situation of each group but also measure the distance between them, enabling the assessment of absolute inequalities without the need for summary inequality indices. Based on this approach, graphs were generated for the three outcomes (early childhood caries, severe early childhood caries and dmft index) to examine inequalities related to socioeconomic position, considering maternal education and family income.

## 3 Results

**FIGURE 1:**
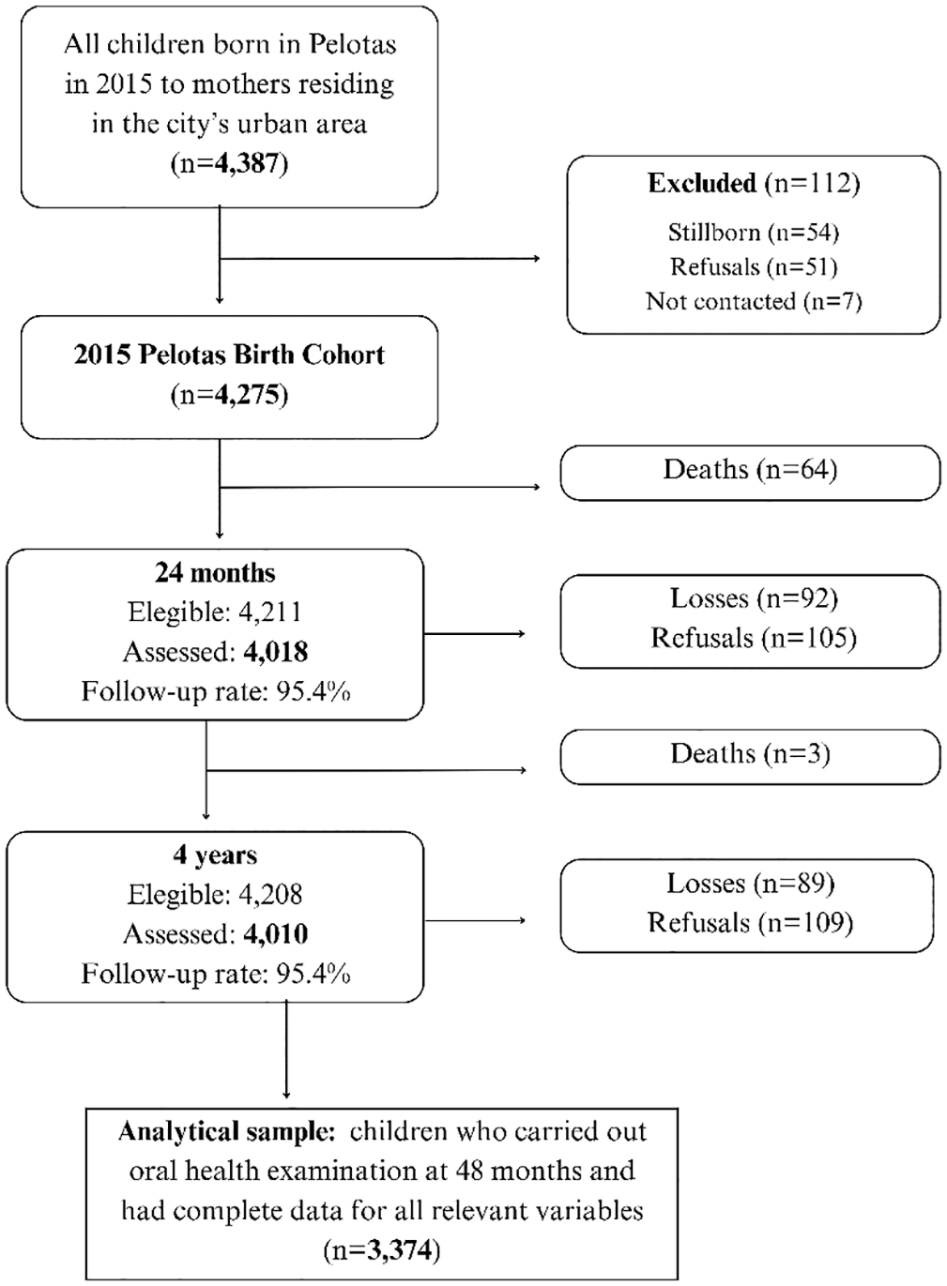
Study participant flowchart. 2015 Pelotas (Brazil) Birth Cohort Study.

From the 4275 eligible participants, 3374 children (50.5% boys and 49.5% girls) carried out the oral health examination and had information on all variables of interest (Figure 1). Socioeconomic and demographic indicators of those children who took part in the oral health study were comparable to those of the complete cohort (Table 1; Table S1). Almost 70% of the mothers had not completed high school (less than 12 years of education) and 63.7% of the families had an income of up to two Brazilian minimum wages. At 24 months, 2474 (73.3%) children were healthy weight (95% CI 71.8; 74.8) and 900 (26.7%) presented overweight/obesity (overweight: 18.8%, 95% CI 17.5; 20.1, and obesity: 7.9%, 95% CI 7.0; 8.9). Regarding caries prevalence at the age of four, 1269 (37.6%) had ECC (95% CI 36.0; 39.2), and 722 (21.4%) severe ECC (95% CI 20.1; 22.8).

**TABLE 1:** Sample descriptive statistics.

	Analytical sample (*n* = 3374)			Full sample (*n* = 4275)		
	*n*	%	95% CI	*n*	%	95% CI
Sex						
Male	1705	50.5	48.8; 52.2	2164	50.6	49.1; 52.1
Female	1669	49.5	47.8; 51.2	2111	49.4	47.9; 50.9
Maternal education, y						
≥ 12	1018	30.2	28.6; 31.7	1330	31.1	29.7; 32.5
9–11	1192	35.3	33.7; 37.0	1458	34.1	32.7; 35.5
5–8	869	25.8	24.3; 27.3	1095	25.6	24.3;27.0
0–4	295	8.7	7.8; 9.7	391	9.2	8.3; 10.0
Maternal age						
< 20	491	14.5	13.4; 15.7	623	14.6	13.5; 15.7
20–34	2393	70.9	69.4; 72.4	3018	70.6	69.2; 72.0
35 or more	490	14.5	13.4; 15.8	633	14.8	13.8; 15.9
Family income						
Up to 2 MW	2148	63.7	62.0; 65.2	2728	63.8	62.4; 65.3
2 or more MW	1226	36.3	34.7; 38.0	1545	36.2	34.7; 37.6
Sugar consumption						
Low	1855	55.0	53.3; 56.6	2202	54.9	53.4; 56.5
High	1519	45.0	43.3; 46.7	1807	45.1	43.5; 46.6
BMI (24 months)						
Healthy weight	2474	73.3	71.8; 74.8	2713	73.0	71.5; 74.4
Overweight	633	18.8	17.5; 20.1	702	18.9	17.6; 20.2
Obese	267	7.9	7.0; 8.9	303	8.1	7.3; 9.1
Outcome (4 years of age)						
Early childhood caries (ECC)						
No	2105	62.4	60.7; 64.0	2283	62.6	61.0; 64.2
Yes	1269	37.6	36.0; 39.2	1362	37.4	35.8; 39.0
Severe ECC (S‐ECC)						
No	2652	78.6	77.2; 79.9	2874	78.8	77.5; 80.1
Yes	722	21.4	20.1; 22.8	771	21.1	19.9; 22.5
dmft (mean; SD; median)	1.0; 2.3; 0			1.0; 2.3; 0		

*Note:* Analytical sample and full sample. BMI 24 months old. 2015 Pelotas (Brazil) Birth Cohort Study.

Among children classified as healthy weight at 24 months, 38.6% (95% CI 36.7; 40.5) presented early childhood caries at the age of four. Among obese children, the prevalence of ECC was 34.8% (95% CI 29.3; 40.7). A similar pattern was observed for severe early childhood dental caries: the prevalence was 22.0% (95% CI 20.4; 23.7) among healthy weight children, 19.9% (95% CI 17.0; 23.2) among overweight, and 19.1% (95% CI 14.8; 24.3) among obese children (Table 2).

**TABLE 2:** Association between BMI at 24 months and dental caries at the age of 4.

	Early childhood caries (*n* = 1269)	Severe early childhood caries (*n* = 722)
	% (95% CI)	% (95% CI)
Body mass index (BMI)		
Healthy weight	38.6 (36.7; 40.5)	22.0 (20.4; 23.7)
Overweight	35.1 (31.4; 38.9)	19.9 (17.0; 23.2)
Obese	34.8 (29.3; 40.7)	19.1 (14.8; 24.3)
Total	37.6 (36.0; 39.2)	21.4 (20.0; 22.8)

*Note:* 2015 Pelotas (Brazil) Birth Cohort Study.

**FIGURE 2:**
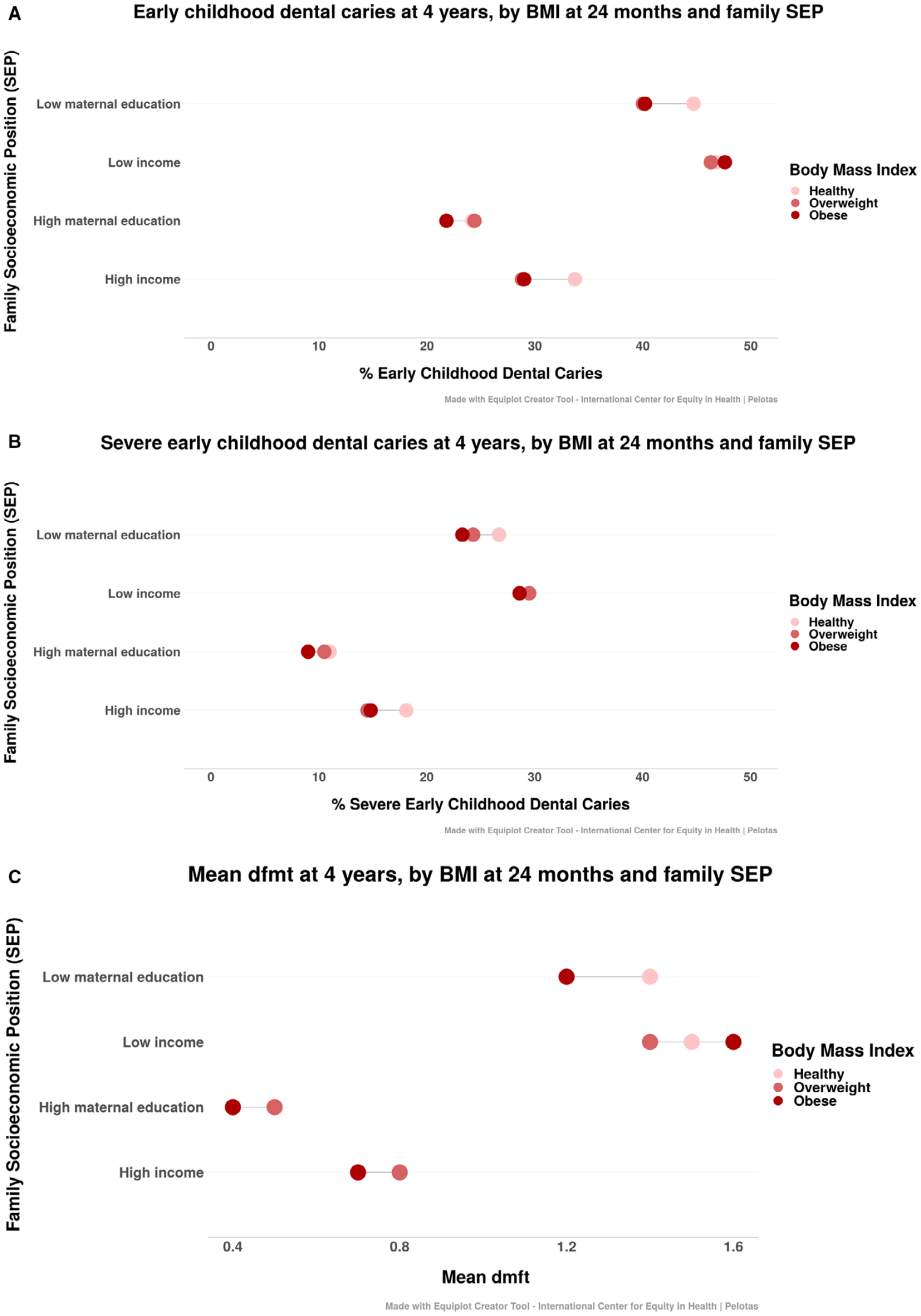
Equiplot graphs illustrating the prevalence of early childhood dental caries at the age of 4 (A), severe early childhood dental caries at the age of 4 (B) and the dmft index at the age of 4 (C), stratified by body mass index (BMI) and family socioeconomic position (SEP). 2015 Pelotas (Brazil) Birth Cohort Study.

Figure 2 displays the Equiplot graphs for the ECC, S‐ECC and dmft index. Children whose mothers have lower levels of education and lower family income show a higher prevalence of these conditions compared to those from more advantageous socioeconomic backgrounds. This data highlights the existence of inequities, both in the occurrence and severity of dental caries. The apparent absence of certain BMI categories in the graphs results from overlapping identical figures for different categories. For example, in the dmft index Equiplot, healthy weight and obese individuals exhibited the same results when high maternal education was considered. These findings are further presented in Table S2 of the Supporting Information, shows BMI at 24 months and dental caries at the age of four, stratified by family income and maternal education during the perinatal period, along with corresponding confidence intervals. In general, children from less educated mothers or poorer families had a higher prevalence of dental caries outcomes, but this was comparable among all nutritional groups. Therefore, no association between nutritional status and dental caries outcomes was identified, overall or within the same socioeconomic strata.

Estimates from the Poisson regression analysis is presented in Table 3. No association was identified between obesity and ECC and S‐ECC. After adjustment for covariates, overweight or obese children presented a prevalence of 6% and 7% lower of early childhood dental caries and severe early childhood caries, respectively, than their peers, but these associations were not statistically significant. In the crude analysis, a significant association was observed between obesity and dmft index. Although overweight and obese children had a 6% lower dmft index in the adjusted analysis, the association was no longer significant (95% CI 0.80; 1.11).

**TABLE 3:** Crude and adjusted association between BMI and dental caries outcomes among participants in the 2015 Pelotas (Brazil) Birth Cohort Study.

	BMI 24 months (*n* = 3374)			
	Crude analysis		Adjusted analysis^a^	
	PR	95% CI	PR	95% CI
Outcome (4 years of age)				
ECC				
Healthy weight	1.00	Reference	1.00	Reference
Overweight/obese	0.91	0.82; 1.00	0.94	0.85; 1.04
S‐ECC				
Healthy weight	1.00	Reference	1.00	Reference
Overweight/obese	0.89	0.77; 1.04	0.93	0.80; 1.08
dmft				
Healthy weight	1.00	Reference	1.00	Reference
Overweight/obese	0.90	0.76; 1.06	0.94	0.80; 1.11

^a^ Adjusted for sex (male, female), maternal age (< 20, 20–34 and ≥ 35), family income (continuous in monthly MW), maternal education at child's birth (continuous in years of formal education), sugar consumption at 24 months (low versus high) and child age (in months) at follow‐up.

## 4 Discussion

The findings of this study indicate no clear or meaningful association between obesity and dental caries in primary dentition. Although the estimates showed no statistically significant relationship, the wide confidence intervals, particularly for the obesity group, suggest some uncertainty and the results should therefore be interpreted with caution. This study adds to the growing body of high‐quality longitudinal evidence on this topic and suggests that, if any association exists, it likely explained by shared risk factors such as sociodemographic and behavioural determinants. These findings emphasise the importance of considering the broader context of child health rather than attributing causal links between these two conditions.

The findings are consistent with previous reports [20, 21]. Ravaghi et al. [20] identified that social deprivation, family structure and ethnicity may moderate the potential association between caries and obesity [20]. Similarly, this study findings align with previous evidence showing that once key confounders, such as diet, socioeconomic status and oral hygiene behaviours, are accounted for, the association tends to disappear [33].

Caries is a multifactorial disease, and any apparent association with obesity is likely due to shared determinants such as high‐sugar diets, socioeconomic inequities and behavioural characteristics [23, 34].

Studies that did not observe such an association suggest that it may become evident as children age, particularly due to the chronic nature of both conditions and the tendency for the prevalence of caries and obesity to increase over time [35, 36]. Some studies also suggest that dietary monitoring among children with obesity may reduce sugar exposure, explaining the null association found [37].

Socioeconomic and environmental contexts remain crucial to understanding both conditions. A recent report about social causes for health inequalities in Brazil highlighted an association between socioeconomic stratification (including income, race, gender and place of living) and health conditions, emphasising that health inequalities are derived from the individual's position in society. Social position, including income, education, continues to be a major determinant of child health [38, 39, 40]. Education affects health on different levels: in the perception of the problem, in the understanding of information about health; in the adoption of healthy behaviours; in the use of health services and in the adherence to therapeutic treatments [41]. A problem with earlier studies is the lack of consistent adjustment for confounders such as race, water fluoridation and access to care [20]. Some systematic reviews recommended that future studies use population‐based samples and standardise the case definitions of BMI and dental caries. They also recommend future studies to adjust for relevant confounding factors, such as sex, diet, oral hygiene habits and socioeconomic status [42, 43, 44].

From a public health perspective, the absence of a direct association underscores the need to address shared risk factors rather than treating these diseases in isolation. Strategies such as taxing sugar‐sweetened foods and reducing socioeconomic inequalities could simultaneously reduce the burden of both conditions.

The high prevalence of obesity in early childhood can reflect a combination of low physical activity, regular consumption of ultra‐processed foods, limited outdoor play, psychosocial stress and genetic factors [45]. Integrated nutrition and oral health programs that target these determinants may provide more sustainable benefits that disease‐specific interventions [46]. In Brazil, community programs offering home visits and dietary counselling during infancy have reduced the incidence of early childhood caries by 22% and its severity by 32% [47]. Encouraging mindful eating and healthier dietary habits can therefore improve both oral and general health [48].

WHO (2004) [29], does not distinguish between fat and lean tissue. This limitation should be acknowledged, although BMI remains a widely accepted and comparable indicator in epidemiological research. Additionally, not all children in the cohort were examined at age four (8.9% were not examined), largely because some interviews were conducted by telephone, video call, or home visits without oral health team follow‐up. Nevertheless, children who missed the clinical examination had sociodemographic characteristics similar to those who were examined, minimising the potential for selection bias.

The present study has several strengths and some limitations. It investigated the association between obesity and dental caries using a longitudinal design, with exposure measured 2 years before the outcome. The study included a large, population‐based sample and achieved a high retention rate, supporting its methodological robustness. However, as in many studies on obesity, the use of BMI percentiles as a measure of body fat, although adjusted for age and sex and allowing comparisons across groups [29], does not distinguish between fat and lean tissue. This limitation should be acknowledged, although BMI remains a widely accepted and comparable indicator in epidemiological research. Additionally, not all children in the cohort were examined at age four (8.9% were not examined), largely because some interviews were conducted by telephone, video call, or home visits without oral health team follow‐up. Nevertheless, children who missed the clinical examination had sociodemographic characteristics similar to those who were examined, minimising the potential for selection bias.

In conclusion, this cohort study of Brazilian children found no strong or consistent association between overweight/obesity and dental caries in the primary dentition. Given the uncertainty of effect estimates, the findings should be interpreted cautiously. Obesity in early childhood should not be considered an independent risk factor for dental caries, but rather a coexisting condition influenced by common social and behavioural determinants.

## Author Contributions

Study conception and planning: Y.M.C., H.S.S., M.S.E. and F.F.D. Manuscript elaboration: Y.M.C., C.F.A. and H.S.S. Data analysis and interpretation: H.S.S. and M.S.E. Manuscript critical review: A.D.B., F.F.D. and G.J.M.G.H. All authors approved the final version of the manuscript and assume public responsibility for its content.

## Funding

This article is based on data from the study “Pelotas Birth Cohort, 2015” conducted by Postgraduate Program in Epidemiology at Universidade Federal de Pelotas, with the collaboration of the Brazilian Public Health Association (ABRASCO). The first phases of the 2015 Pelotas (Brazil) Birth Cohort was funded by the Wellcome Trust (095582). Funding for specific follow‐up visits was also received from the Conselho Nacional de Desenvolvimento Científico e Tecnológico (CNPq) and Fundação de Amparo a Pesquisa do Estado do Rio Grande do Sul (FAPERGS) and Children's Pastorate sponsored follow‐up at 24 months; and FAPERGS—PPSUS, the Wellcome Trust (10735_Z_18_Z) and the Bernard van Leer Foundation (BRA‐2018‐178) for the 48 months follow‐up.

## Ethics Statement

This study was approved by the Research Ethics Committee of the Federal University of Pelotas under protocol number 31296614.1.0000.5317. Additionally, all surveys conducted at the different follow‐ups, including the oral health study, received approval from the Ethics Committee of the School of Medicine at the Federal University of Pelotas.

## Conflicts of Interest

The authors declare no conflicts of interest.

## Supporting information


**Table S1:** Sample descriptive statistics. Analytical sample and Full sample. BMI 24 months old. 2015 Pelotas (Brazil) Birth Cohort Study.
**Table S2:** BMI at 24 months and dental caries at the age of 4, stratified by family income and maternal education at perinatal. 2015 Pelotas (Brazil) Birth Cohort Study (*n* = 3374).

## Data Availability

The data that support the findings of this study are available on request from the corresponding author. The data are not publicly available due to privacy or ethical restrictions.
