# Supplementary material for: Is Obesity Associated With Dental Caries in Primary Dentition? Findings From a Birth Cohort in Southern Brazil
Source: Community Dent Oral Epidemiol. 2026 Feb 20;54(4):427–35. doi: 10.1111/cdoe.70056 (PMC13428047; doi:10.1111/cdoe.70056)
Supplement: Supplementary file 1 — Table S1: Sample descriptive statistics. Analytical sample and Full sample. BMI 24 months old. 2015 Pelotas (Brazil) Birth Cohort Study. Table S2: BMI at 24 months and dental caries at the age of 4, stratified by family income and maternal education at perinatal. 2015 Pelotas (Brazil) Birth Cohort Study (n = 3374). [file CDOE-54-427-s001.docx]

**SUPPLEMENTARY FILE**

**Is obesity associated with dental caries in primary dentition? Findings from a Birth Cohort in Southern Brazil**

Yorrana Martins Corrêa^1^ https://orcid.org/0000-0003-1568-783X

Cinthia Fonseca Araujo^2^ <https://orcid.org/0000-0002-5148-4274>

Mariana Silveira Echeverria^2^ <https://orcid.org/0000-0002-2045-4232>

Andréa Dâmaso Bertoldi^2^ <https://orcid.org/0000-0002-4680-3197>

Geert JMG van der Heijden^3^ <https://orcid.org/0000-0003-4979-2099>

Flávio Fernando Demarco^1,2^ <https://orcid.org/0000-0003-2276-491X>

Helena Silveira Schuch^1,4^ https://orcid.org/0000-0001-9932-9698

^1^Graduate Program in Dentistry, Federal University of Pelotas, Gonçalves Chaves St, 457 – Centro, CEP 96015-560, Pelotas, RS, Brazil.

^2^Graduate Program in Epidemiology, Federal University of Pelotas, Marechal Deodoro St, 1160 – Centro, CEP 96020-220, Pelotas, RS, Brazil.

^3^Oral Public Health Department, ACTA, Academic Center for Dentistry, University of Amsterdam - Amsterdam, Netherlands.

^4^Population Oral Health, School of Dentistry, The University of Queensland. Herston, Australia.

**Corresponding author:**

Helena Silveira Schuch, Herston Road 288, 4006, Herston, QLD, Australia. E-mail: [h.schuch@uq.edu.au](mailto:h.schuch@uq.edu.au); helenasschuch@gmail.com

Table S1. Sample descriptive statistics. Analytical sample and Full sample. BMI 24 months old. 2015 Pelotas (Brazil) Birth Cohort Study.

|  | Analytical sample (n=3,374) | | | Full sample (n=4,275) | | |
| --- | --- | --- | --- | --- | --- | --- |
|  | n | % | 95% CI | n | % | 95% CI |
| **Sex** |  |  |  |  |  |  |
| Male | 1,705 | 50.5 | 48.8; 52.2 | 2,164 | 50.6 | 49.1; 52.1 |
| Female | 1,669 | 49.5 | 47.8; 51.2 | 2,111 | 49.4 | 47.9; 50.9 |
| Missing | NA | - | - | 0 | - | - |
| **Maternal education, y** |  |  |  |  |  |  |
| ≥12 | 1,018 | 30.2 | 28.6; 31.7 | 1,330 | 31.1 | 29.7; 32.5 |
| 9-11 | 1,192 | 35.3 | 33.7; 37.0 | 1,458 | 34.1 | 32.7; 35.5 |
| 5-8 | 869 | 25.8 | 24.3; 27.3 | 1,095 | 25.6 | 24.3;27.0 |
| 0-4 | 295 | 8.7 | 7.8; 9.7 | 391 | 9.2 | 8.3; 10.0 |
| Missing | NA | - | - | 1 | 0.02 | <0.001; 1.7 |
| **Maternal age** |  |  |  |  |  |  |
| <20 | 491 | 14.5 | 13.4; 15.7 | 623 | 14.6 | 13.5; 15.7 |
| 20-34 | 2,393 | 70.9 | 69.4; 72.4 | 3,018 | 70.6 | 69.2; 72.0 |
| 35 or more | 490 | 14.5 | 13.4; 15.8 | 633 | 14.8 | 13.8; 15.9 |
| Missing | NA | - | - | 1 | 0.02 | <0.001; 1.7 |
| **Family income** |  |  |  |  |  |  |
| Up to 2 MW | 2,148 | 63.7 | 62.0; 65.2 | 2,728 | 63.8 | 62.4; 65.3 |
| 2 or more MW | 1,226 | 36.3 | 34.7; 38.0 | 1,545 | 36.2 | 34.7; 37.6 |
| Missing | NA | - | - | 2 | 0.05 | 0.01; 1.9 |
| **Sugar consumption** |  |  |  |  |  |  |
| Low | 1,855 | 55.0 | 53.3; 56.6 | 2,202 | 54.9 | 53.4; 56.5 |
| High | 1,519 | 45.0 | 43.3; 46.7 | 1,807 | 45.1 | 43.5; 46.6 |
| Missing | NA | - | - | 266 | 6.2 | 5.5; 7.0 |
| **BMI (24 months)** |  |  |  |  |  |  |
| Healthy weight | 2,474 | 73.3 | 71.8; 74.8 | 2,713 | 73.0 | 71.5; 74.4 |
| Overweight | 633 | 18.8 | 17.5; 20.1 | 702 | 18.9 | 17.6; 20.2 |
| Obese | 267 | 7.9 | 7.0; 8.9 | 303 | 8.1 | 7.3; 9.1 |
| Missing | NA | - | - | 557 | 13.0 | 12.0; 14.1 |
| Outcome (4 years of age) |  |  |  |  |  |  |
| **Early childhood caries (ECC)** |  |  |  |  |  |  |
| No | 2,105 | 62.4 | 60.7; 64.0 | 2,283 | 62.6 | 61.0; 64.2 |
| Yes | 1,269 | 37.6 | 36.0; 39.2 | 1,362 | 37.4 | 35.8; 39.0 |
| Missing | NA | - | - | 630 | 14.7 | 13.7; 15.8 |
| **Severe ECC (S-ECC)** |  |  |  |  |  |  |
| No | 2,652 | 78.6 | 77.2; 79.9 | 2,874 | 78.8 | 77.5; 80.1 |
| Yes | 722 | 21.4 | 20.1; 22.8 | 771 | 21.1 | 19.8; 22.5 |
| Missing | NA | - | - | 630 | 14.7 | 13.7; 15.8 |
| **DMFT (mean; SD; median)** | 1.0; 2.3; 0 | | | 1.0; 2.3; 0 | | |

| Table S2. BMI at 24 months and dental caries at the age of 4, stratified by family income and maternal education at perinatal. 2015 Pelotas (Brazil) Birth Cohort Study. (n=3,374) | | | | | | | | |
| --- | --- | --- | --- | --- | --- | --- | --- | --- |
|  | BMI 24 months | | | | | | | |
|  | Healthy weight | | | | Overweight | | Obese | |
|  | n | % | | n | | % | n | % |
| Outcome (4 years of age) | | | **High maternal education** | | | | | |
| **ECC** |  |  | |  | |  |  |  |
| No | 560 | 75.8 (72.6; 78.7) | | 152 | | 75.6 (69.2; 81.1) | 61 | 78.2 (67.7; 86.0) |
| Yes | 179 | 24.2 (21.3; 27.4) | | 49 | | 24.4 (18.9; 30.8) | 17 | 21.8 (14.0; 32.3) |
|  | **Low maternal education** | | | | | | | |
| **ECC** |  |  | |  | |  |  |  |
| No | 960 | 55.3 (53.0; 57.7) | | 259 | | 60.0 (55.2; 64.5) | 113 | 59.8 (52.6; 66.5) |
| Yes | 775 | 44.7 (42.3; 47.0) | | 173 | | 40.0 (35.5; 44.7) | 76 | 40.2 (33.5; 47.4) |
|  | **High family income** | | | | | | | |
| **ECC** |  |  | |  | |  |  |  |
| No | 1,033 | 66.3 (63.9; 68.6) | | 289 | | 71.2 (66.6; 75.4) | 130 | 71.0 (64.0; 77.1) |
| Yes | 526 | 33.7 (31.4; 36.1) | | 117 | | 28.8 (24.6; 33.4) | 53 | 29.0 (22.8; 35.9) |
|  | **Low family income** | | | | | | | |
| **ECC** |  |  | |  | |  |  |  |
| No | 487 | 53.2 (50.0; 56.4) | | 122 | | 53.7 (47.2; 60.1) | 44 | 52.4 (41.7; 62.8) |
| Yes | 428 | 46.8 (43.6; 50.0) | | 105 | | 46.3 (39.9; 52.8) | 40 | 47.6 (37.2; 58.3) |
|  | **High maternal education** | | | | | | | |
| **S-ECC** |  |  | |  | |  |  |  |
| No | 658 | 89.0 (86.6; 91.1) | | 180 | | 89.5 (84.5; 93.1) | 71 | 91.0 (82.3; 95.7) |
| Yes | 81 | 11.0 (8.9; 13.4) | | 21 | | 10.5 (6.9; 15.5) | 7 | 9.0 (4.3; 17.6) |
|  | **Low maternal education** | | | | | | | |
| **S-ECC** |  |  | |  | |  |  |  |
| No | 1,271 | 73.3 (71.1; 75.3) | | 327 | | 75.7 (71.4; 79.5) | 145 | 76.7 (70.2; 82.2) |
| Yes | 464 | 26.7 (24.7; 28.9) | | 105 | | 24.3 (20.5; 28.6) | 44 | 23.3 (17.8; 29.8) |
|  | **High family income** | | | | | | | |
| **S-ECC** |  |  | |  | |  |  |  |
| No | 1,277 | 81.9 (79.9; 83.7) | | 347 | | 85.5 (81.7; 88.6) | 156 | 85.2 (79.3; 89.7) |
| Yes | 282 | 18.1 (16.2; 20.1) | | 59 | | 14.5 (11.4; 18.3) | 27 | 14.8 (10.3; 20.7) |
|  | **Low family income** | | | | | | | |
| **S-ECC** |  |  | |  | |  |  |  |
| No | 652 | 71.3 (68.2; 74.1) | | 160 | | 70.5 (64.2; 76.1) | 60 | 71.4 (60.9; 80.1) |
| Yes | 263 | 28.7 (25.9; 31.8) | | 67 | | 29.5 (23.9; 35.8) | 24 | 28.6 (19.9; 39.1) |
|  | **High maternal education** | | | | | | | |
|  | Mean | (SD) | | Mean | | (SD) | Mean | (SD) |
| DMFT | 0.4 | (1.3) | | 0.5 | | (1.4) | 0.4 | (1.1) |
|  | **Low maternal education** | | | | | | | |
| DMFT | 1.4 | (2.6) | | 1.2 | | (2.3) | 1.2 | (2.4) |
|  | **High family income** | | | | | | | |
| DMFT | 0.8 | (2.0) | | 0.8 | | (1.8) | 0.7 | (1.6) |
|  | **Low family income** | | | | | | | |
| DMFT | 1.5 | (2.8) | | 1.4 | | (2.4) | 1.6 | (2.8) |
| Maternal education: Up to incomplete high school versus completed high school or more. | | | | | | | | |
| Family income: Less than 2 minimum wages (MW) versus 2 or more MW. | | | | | | | | |
